# Neural and behavioural indices of face processing in siblings of children with autism spectrum disorder (ASD): A longitudinal study from infancy to mid-childhood

**DOI:** 10.1016/j.cortex.2020.02.008

**Published:** 2020-06

**Authors:** Elizabeth Shephard, Bosiljka Milosavljevic, Luke Mason, Mayada Elsabbagh, Charlotte Tye, Teodora Gliga, Emily JH. Jones, Tony Charman, Mark H. Johnson, The BASIS Team, Simon Baron-Cohen, Rachael Bedford, Patrick Bolton, Susie Chandler, Janice Fernandes, Holly Garwood, Kristelle Hudry, Greg Pasco, Andrew Pickles, Leslie Tucker, Agnes Volein

**Affiliations:** a Department of Child & Adolescent Psychiatry, Institute of Psychiatry, Psychology & Neuroscience, King's College London, UK; b Department of Psychology, Institute of Psychiatry, Psychology & Neuroscience, King's College London, UK; c Centre for Brain and Cognitive Development, Birkbeck, University of London, UK; d Montreal Neurology Institute and Hospital, McGill University, Canada; e University of East Anglia, Norwich, UK; f Department of Psychology, Cambridge University, UK

**Keywords:** Autism spectrum disorder (ASD), Infant siblings, Face processing, EEG, Development

## Abstract

Impaired face processing is proposed to play a key role in the early development of autism spectrum disorder (ASD) and to be an endophenotypic trait which indexes genetic risk for the disorder. However, no published work has examined the development of face processing abilities from infancy into the school-age years and how they relate to ASD symptoms in individuals with or at high-risk for ASD. In this novel study we investigated neural and behavioural measures of face processing at age 7 months and again in mid-childhood (age 7 years) as well as social-communication and sensory symptoms in siblings at high (*n* = 42) and low (*n* = 35) familial risk for ASD. In mid-childhood, high-risk siblings showed atypical P1 and N170 event-related potential correlates of face processing and, for high-risk boys only, poorer face and object recognition ability compared to low-risk siblings. These neural and behavioural atypicalities were associated with each other and with higher social-communication and sensory symptoms in mid-childhood. Additionally, more atypical neural correlates of object (but not face) processing in infancy were associated with less right-lateralised (more atypical) N170 amplitudes and greater social-communication problems in mid-childhood. The implications for models of face processing in ASD are discussed.

## 1 Introduction

Autism spectrum disorder (ASD) is a neurodevelopmental condition characterised by social-communication impairments, restricted and repetitive behaviours and sensory atypicalities (American Psychiatric Association, 2013). In addition to the core symptoms of ASD, many individuals experience cognitive impairments, including atypicalities in processing social stimuli such as faces. For instance, children and adults with ASD show poorer recognition memory for faces and reduced visual attention to face stimuli compared to typically developing individuals (Guillon et al., 2014, Neuhaus et al., 2015, Webb et al., 2017, Weigelt et al., 2012, Weigelt et al., 2013). Neurophysiological correlates of face processing such as the P1 and N170 event-related potential (ERP) components have also been found to be atypical in children and adults with ASD (Kovarski et al., 2019, Neuhaus et al., 2015, Tye et al., 2013, see also; Kang et al., 2018, Sysoeva et al., 2018).

The P1 is a positive-going deflection in the electro-encephalographic (EEG) or magneto-encephalographic (MEG) waveform that is maximal at occipital regions around 100 msec post-stimulus and is believed to index early visual attention; specifically, the P1 is faster and/or larger for face versus non-face stimuli, which may reflect enhanced attentional orienting to these important stimuli and/or the process of identifying a stimulus as “face-like” early in visual processing (Itier and Taylor, 2004, Rossion et al., 1999). Following the P1, the N170 is a negative-going deflection that is maximal ∼170 msec post-stimulus at temporal-parietal scalp regions (Bentin, Allison, Puce, Perez, & McCarthy, 1996). The N170 is larger over the right than left hemisphere, larger and faster for face than non-face stimuli, and slower and/or larger when configural information concerning spatial relationships between facial features is disrupted (as is the case for inverted faces, for example); as such this component is thought to reflect early face-selective processes including extraction of configural information and categorisation of a stimulus as a face (Bentin et al., 1996, Ince et al., 2016; Itier & Taylor, 2004). In ASD, reduced right-hemisphere lateralisation of the N170 (Kovarski et al., 2019, Tye et al., 2013), slowed P1 (Neuhaus et al., 2016) and N170 (Kang et al., 2018, Sysoeva et al., 2018) latencies, reduced N170 amplitude (Kovarski et al., 2019) and increases (Batty, Meaux, Wittemeyer, Rogé, & Taylor, 2011) or decreases in P1 amplitudes (Kovarski et al., 2019) have been reported, suggestive of alterations in neural mechanisms underlying face processing.

These atypicalities in face recognition, attention to faces and neurophysiological correlates of face processing have been shown to correlate with more severe ASD symptoms and poorer social functioning (Kovarski et al., 2019, Neuhaus et al., 2016, Neuhaus et al., 2015, Tye et al., 2013, Weigelt et al., 2013), indicating that altered face processing abilities are associated with ‘real-world’ social deficits and ASD symptoms. Poorer face memory and altered neurophysiological correlates of face processing have also been reported in unaffected siblings and parents of individuals with ASD, suggesting that impaired face processing might represent an endophenotypic trait which indexes genetic risk for the disorder (Dawson et al., 2005a, de Klerk et al., 2014, Wallace et al., 2010; but see; Anzures et al., 2016, Sysoeva et al., 2018). Furthermore, several models propose that altered face processing plays a key role in the development of ASD. Fundamental to these models is the premise that altered face processing in the first months of life restricts the development of specialised cortical face processing systems and leads to impairments in social-communication behaviours that rely on efficient processing of information gleaned from faces.

Specifically, *perceptual/cognitive* models propose that innate differences in the neural regions underlying face processing (e.g., fusiform gyrus) compromise processing in ASD, for example by preventing the extraction of perceptual and affective information from faces, resulting in difficulties using this information for social cognition and communication (see Dawson et al., 2005b, Schultz, 2005). In turn, these difficulties may make social interactions less rewarding and lead to decreases in social attention and poorer social learning (Dawson et al., 2005b, Schultz, 2005). In contrast, *social attention* models assert that early decreases in attention to social stimuli in ASD reduce opportunities to learn about faces and thereafter lead to downstream differences in face processing abilities (see Dawson et al., 2005b, Webb et al., 2017). Important for both accounts of face processing atypicalities in ASD is Johnson's (2000, 2011) *interactive specialisation* framework, which is based on empirical work in typically developing children and indicates that face processing continues to improve throughout childhood and adolescence, with neurocognitive systems becoming increasingly specialised for face processing as the child seeks and acquires greater experience with faces. In ASD, early face processing atypicalities, caused either by perceptual/cognitive alterations (Dawson et al., 2005b, Schultz, 2005) or decreased social attention (Dawson et al., 2005b, Webb et al., 2017), may hinder the experience-dependent specialisation of this ability and, consequently, deficits may become compounded over time.

Prospective longitudinal studies investigating face processing in relation to the emergence of ASD symptoms in infants at high familial risk for ASD have provided the opportunity to test these models. These ‘high-risk’ infants are younger-born siblings of children with ASD and are ∼20 times more likely to develop ASD themselves than infants without an older sibling with ASD (Messinger et al., 2015, Ozonoff et al., 2011). A further 20–30% of high-risk infants develop subclinical ASD traits (Charman et al., 2017, Messinger et al., 2013). Several studies have reported hemispheric atypicalities in neurophysiological correlates of face processing, including reduced right-hemisphere lateralisation of the P1 and N290/P400 (infant precursors of the N170) ERP components and increased left-hemisphere lateralisation of oscillatory gamma activity, in high-risk infants compared to low-risk infants during the first year of life (Keehn et al., 2015, McCleery et al., 2009; see also; Guy et al., 2018, Luyster et al., 2014). These findings might indicate that the early development of right-lateralised face processing is disrupted in infants with familial risk for ASD. Atypicalities in other ERP markers of face processing (P400 and Nc components) indicative of reduced attention to face stimuli have also been reported in the first year of life, although these were restricted to high-risk infants who later (at age 2 years) met diagnostic criteria for ASD and were not present in high-risk infants without later ASD (Jones et al., 2016). Similarly, eye-tracking studies have revealed reduced visual attention to face stimuli in high-risk infants who met diagnostic criteria for ASD at age 2–3 years compared to high-risk infants who did not develop ASD and low-risk infants (Chawarska et al., 2013, Jones et al., 2016). Other studies, however, found increased attention to faces in high-risk infants compared to low-risk infants but this was not associated with ASD outcomes (Elsabbagh et al., 2013). In summary, consistent with both social attention and perceptual/cognitive models, atypicalities in visual attention to and neural processing of faces have been found in the first year of life in infants at high familial risk for ASD, although there is some heterogeneity in the specific alterations observed and in the extent to which these index familial risk for ASD or are restricted to infants who develop ASD.

Taken together, the findings from studies of high-risk infants, older individuals with ASD and their siblings and parents suggest that face processing atypicalities are present early in infancy and persist into childhood and adulthood in individuals with ASD and in those with familial risk for ASD. In support, one longitudinal study that collected measures of face processing in infancy (Elsabbagh et al., 2013) and again at age 3 years (de Klerk et al., 2014) reported that high-risk infants with more atypical visual attention to face stimuli (in this sample, increased attention to faces) showed the poorest face recognition ability at age 3, indicating that early disruptions to face processing persist into early childhood. However, to our knowledge no published longitudinal data has shown whether face processing atypicalities in high-risk infants persist longer-term, beyond early childhood and into the school-age years. Investigating the longer-term development of face processing in these infants is important because deficits may worsen (or improve) over time with experience-dependent specialisation of the face processing system (Johnson, 2000, 2011). Indeed, Webb et al. (2017) note that in mid-childhood, typically developing children show a marked improvement in face processing ability and that performance and neural indices become close to adult-like during this period; in children with ASD, however, face processing development is delayed and impairments become more pronounced during mid-childhood, although this suggestion was based on cross-sectional rather than longitudinal data. In addition, no published longitudinal work has investigated the developmental trajectories of both behavioural measures (e.g., visual attention to faces and face recognition ability) and neural correlates of face processing from infancy into childhood in the same sample of individuals with or at risk for ASD. Such investigations are needed to fully understand the nature and development of face processing atypicalities in ASD and high-risk for ASD. It is important to know, for example, whether behaviourally-observed alterations in visual attention to faces and face recognition ability represent the same or different form of impairment to atypicalities in neural function measured during face viewing tasks, and whether early social attention atypicalities (or atypicalities in neural correlates of face processing) associate with both later neural processing of faces and behavioural face recognition ability.

In the current study we aimed to address these issues by examining, for the first time, the development of face processing in a sample of siblings at high and low familial risk for ASD followed longitudinally from the first year of life into mid-childhood. Face processing was assessed at age 7 months using eye-tracking measures of attention to face stimuli and neurophysiological correlates of face processing; we previously reported on these infant measures in relation to ASD outcomes at age 3 years and found atypical ERP indices of face processing in high-risk infants who met diagnostic criteria for ASD (Elsabbagh et al., 2012; Tye et al., In submission) and atypically increased attention to face stimuli in high-risk infants that was independent of ASD outcomes (Elsabbagh et al., 2013) and was associated with poorer face recognition ability in early childhood (de Klerk et al., 2014). Face processing was measured again at age 7 years with behavioural measures of face recognition ability and neurophysiological correlates of face processing.

We conducted cross-sectional and longitudinal analyses (summarised in Table 1) to better understand the nature of face processing difficulties in children at high-risk for ASD, including those with ASD diagnoses, and the developmental trajectory of face processing in ASD and high-risk for ASD. In particular, we addressed the following research questions and hypotheses:

**Table 1:** Summary of study research questions, hypotheses and methods.

Research question	Hypothesis	Measures	Statistical analysis
1. Cross-sectionally, do HR and LR siblings differ in face processing abilities in mid-childhood and are atypicalities in HR siblings driven by the subset of children with ASD?	HR siblings, regardless of ASD diagnosis, would show poorer face recognition performance and atypical neural correlates of face processing compared to LR siblings.	In mid-childhood, face processing was measured behaviourally with performance on the face recognition task (accuracy and RT for faces, bodies, cars and scenes) and at the neural level with neurophysiological correlates of upright and inverted face processing (P1 and N170 amplitudes and latencies to upright and inverted faces).	*Face recognition:* 2 (group: HR, LR) *x* 4 (condition: faces, cars, bodies, scenes) ANOVAs for accuracy and RT *Neural correlates of face processing:* 2 (group: HR, LR) *x* 2 (condition: upright, inverted) *x* 2 (hemisphere: left, right) ANOVAs for P1/N170 amplitude/latency *Supplementary analysis:* All models above repeated including age and IQ as covariates. ANOVA models above repeated with ASD-group (HR-ASD, HR-non-ASD, LR) in place of HR/LR group to assess whether HR-ASD group driving effects.
2. How are atypicalities in face processing associated with each other? In particular, longitudinally, do atypicalities in face processing in HR infants associate with face processing abilities in mid-childhood? Cross-sectionally, do high-risk children with the most atypical neural correlates of face processing show the poorer face recognition ability?	HR infants with the most atypical face processing abilities would show the poorest face recognition and most atypical neural correlates of face processing in mid-childhood. In mid-childhood, greater atypicality in neural correlates of face processing would be associated with poorer face recognition ability.	Face processing was measured in infancy (at age 7 months) in terms of visual attentional engagement with face stimuli in the Pop-out task and at the neural level by the N290 amplitude difference score for viewing face versus noise stimuli. Face processing in mid-childhood was measured by recognition accuracy and RT to face trials in the face recognition task and at the neural level by the P1 latency inversion effect and N170 amplitude lateralisation index.	*Longitudinal associations:* Spearman correlation coefficients computed between attentional engagement with faces/N290 difference scores in infancy and mid-childhood face recognition accuracy/RT, P1 latency inversion effect and N170 lateralisation index. *Cross-sectional associations:* Spearman correlation coefficients were computed between mid-childhood face recognition accuracy and RT and the mid-childhood P1 latency inversion effect and N170 lateralisation index.
3. How do face processing abilities relate to clinical and subclinical ASD symptoms (social-communication impairments and sensory processing atypicalities) in HR siblings?	In mid-childhood and longitudinally, face processing atypicalities would be associated with more severe social-communication impairments and fewer sensory processing atypicalities.	Face processing measures in infancy (visual attentional engagement with faces; N290 amplitude difference score) and in mid-childhood (recognition accuracy and RT for face trials; P1 latency inversion effect and N170 lateralisation index) were associated with mid-childhood social-communication and sensory processing symptoms (measured by the SRS-2 and SSP, respectively).	Spearman correlation coefficients were computed between infant and mid-childhood face processing measures and mid-childhood social-communication and sensory symptom scores.

*HR* = high-risk siblings, *LR* = low-risk siblings. *HR-ASD/HR-non-ASD* = high-risk siblings who did (HR-ASD) and did not (HR-non-ASD) meet diagnostic criteria for ASD in mid-childhood. *N290 amplitude difference score* = N290 amplitude for faces – N290 amplitude for noise stimuli. *Visual attentional engagement* = proportion of time the infants spent looking at the face image compared to the object images in the Face Pop-out task. *P1 latency inversion effect* = extent to which P1 latencies were longer for inverted than upright faces. *N170 lateralisation index* = extent to which N170 amplitude was larger in the right than left hemisphere. *SRS-2* = Social Responsiveness Scale – Revised. *SSP* = Short Sensory Profile.

Question/Hypothesis 1: Cross-sectionally, do high-risk and low-risk siblings differ in face processing abilities in mid-childhood and are atypicalities in high-risk siblings driven by the subset of children with ASD? Following previous research (de Klerk et al., 2014), we predicted that high-risk siblings, regardless of ASD diagnosis, would show poorer face recognition performance and atypical neural correlates of face processing compared to low-risk siblings.

Question/Hypothesis 2: How are atypicalities in face processing associated with each other? In particular, longitudinally, do atypicalities in face processing in high-risk infants associate with face processing abilities in mid-childhood? We predicted that high-risk infants with the most atypical face processing abilities would show the poorest face recognition and most atypical neural correlates of face processing in mid-childhood. Cross-sectionally, do high-risk children with the most atypical neural correlates of face processing show the poorest face recognition ability? We predicted that greater atypicality in neural correlates of face processing would be associated with poorer face recognition ability.

Question/Hypothesis 3: How do face processing abilities relate to clinical and subclinical ASD symptoms in high-risk siblings? To address this question, we examined associations between infant and mid-childhood face processing indices and the severity of mid-childhood social-communication impairments and sensory processing atypicalities. Based on previous work (Kovarski et al., 2019, Neuhaus et al., 2016, Neuhaus et al., 2015, Tye et al., 2013, Weigelt et al., 2013) we predicted that face processing atypicalities would be associated with more severe social-communication impairments. In contrast, we predicted that greater face processing atypicalities might be associated with *fewer* sensory processing atypicalities. While this hypothesis may appear contradictory, recent work indicates that sensory symptoms might *enhance* rather than impair face processing abilities in high-risk infants and toddlers with ASD because hyper-sensitivity to incoming information, including social stimuli, may result in enhanced attention to and subsequent processing of such stimuli (Jones, Dawson, & Webb, 2018).

## 2 Methods

### 2.1 Participants

Participants were 104 children taking part in a prospective longitudinal study of infants at high- and low- familial risk for autism (hereafter, HR and LR) recruited as part of the British Autism Study of Infant Siblings (BASIS; www.basisnetwork.org). Siblings completed research visits at 7 and 14 months of age, around their second and third birthdays, and were invited to return for a follow-up study at age 7 years. At enrolment, each HR infant (*n* = 54) had an older sibling (in 4 cases, a half-sibling) with a community clinical ASD diagnosis, confirmed using information from the *Development and Well-Being Assessment* (*DAWBA*; Goodman, Ford, Richards, Gatward, & Meltzer, 2000) and the *Social Communication Questionnaire* (*SCQ*; Rutter, Bailey, & Lord, 2003) by expert clinicians (TC, PB).^2^ Parent-reported family medical histories were examined for significant conditions in the proband or extended family members (e.g., Fragile X syndrome, tuberous sclerosis) with no such conditions reported. LR controls (*n* = 50) were full-term infants (one exception) recruited from a volunteer database at the Birkbeck Centre for Brain and Cognitive Development. Medical history review confirmed lack of ASD within first-degree relatives. At enrolment, all LR infants had at least one older sibling. The SCQ was used to confirm absence of ASD in these older siblings, with no child scoring above instrument cut-off (≥15; *n* = 1 missing data). Not all children were retained at the 7-year assessment (44 HR and 37 LR participated; see Supplementary Materials for retention analysis). Of these, two HR children and two LR children did not complete a research visit (parents completed questionnaires only) and were excluded from the current analysis, leaving a final sample of 42 HR siblings and 35 LR controls (see Table 2 for group characteristics). Ethical approval was obtained from the NHS National Research Ethics Service (NHS RES London REC 08/H0718/76; 14/LO/0170). Parents provided written informed consent at all visits. Children provided written informed assent at the mid-childhood visit if possible given developmental level.

**Table 2:** Characteristics of the HR and LR groups at the 7-month and 7-year assessments. Means (SD) are presented.

	HR group (*n* = 42)	LR group (*n* = 35)	Group differences
Sex (n girls, n boys)	27, 15	21, 14	n/s
**Mid-childhood (7-year) measures of ASD and face processing**			
Age (months) *N (girls)*	90.57 (6.20) *42 (27)*	89.34 (4.81) *35 (21)*	n/s
SRS-2 T-score *N (girls)*	60.11 (19.81) *35 (22)*	45.52 (5.92) *33 (20)*	*t* (40.35) = −4.17, *p* < .001, *d* = .94
SSP Total score *N (girls)*	159.67 (29.82) *36 (22)*	173.78 (11.76) *32 (20)*	*t* (46.70) = 2.62, *p* = .01, *d* = .62
WASI-II FSIQ *N (girls)*	109.34 (16.29) *41 (27)*	117.06 (11.61) *35 (21)*	*t* (74) = 2.34, *p* = .02, *d* = .55
EEG face processing unattended trials *N (girls)*	13.00 (14.24) *19 (12)*	6.25 (12.76) *28 (16)*	n/s
Upright face trials for analysis *N (girls)*	74.11 (7.91) *19 (12)*	75.54 (11.99) *28 (16)*	n/s
Inverted face trials for analysis *N (girls)*	78.21 (6.78) *19 (12)*	80.71 (11.34) *28 (16)*	n/s
**Infant (7-month) measures of face processing**			
Age (months) *N (girls)*	7.43 (1.23) *42 (27)*	7.29 (1.15) *35 (21)*	n/s
N290 amplitude difference (μv) *N (girls)*	−5.44 (7.38) *33 (20)*	−7.44 (5.89) *23 (14)*	–
N290 latency difference (ms) *N (girls)*	8.08 (18.40) *33 (20)*	8.54 (24.50) *23 (14)*	–
Face versus object looking time *N (girls)*	.46 (.14) *40 (26)*	.44 (.13) *30 (20)*	–

*SRS-2 =* Social Responsiveness Scale – Revised (higher scores = greater social-communication impairments). *SSP =* Short Sensory Profile (lower scores = greater sensory symptoms). *WASI-II FSIQ =* Wechsler Abbreviated Scale of Intelligence – 2nd Edition full-scale intelligence quotient. *EEG face processing unattended trials =* Number of trials excluded due to participant not attending to the screen. *Upright/Inverted face trials for analysis =* Number of upright and inverted face trials remaining for analysis after exclusions due to inattention and artefacts. *N290 amplitude difference =* difference score representing the extent to which amplitude of the N290 ERP component was larger (more negative) for face than for noise stimuli. *N290 latency difference* = difference score representing the extent to which N290 latency was longer for face than noise stimuli. *Face versus object looking time =* proportion of time spent looking at face images versus object Pop-Out array images.

### 2.2 Assessments of ASD symptoms and face processing in mid-childhood

#### 2.2.1 ASD symptoms and IQ

A battery of assessments was used to measure ASD symptoms and assign research diagnoses of ASD (see Supplementary Materials for full details). Fifteen HR children met DSM-5 criteria for ASD and the remaining 27 HR and 35 LR children did not. Parent-report measures of social-communication and sensory symptoms were used in the current analyses. The *Social Responsiveness Scale – Second Edition* (*SRS-2*; Constantino, 2012) assessed parent-rated social impairments over the 6 months prior to testing and age-and sex-normed T-scores (mean 50; SD 10) were used in analysis. The *Short Sensory Profile* (*SSP*; Dunn, 1999) assessed parent-rated sensory processing difficulties; the total SSP score was used in analysis. Higher scores on the SRS-2 reflect more severe social-communication impairments; lower scores on the SSP reflect greater sensory processing problems. The *Wechsler Abbreviated Scale of Intelligence – Second Edition* (*WASI-II*; Wechsler, 2011), a standardised instrument to assess intellectual ability, was completed with each child. Age-normed intelligence quotients (mean 100; SD 15) for full-scale IQ (FSIQ) were used in analysis. One HR child was unable to complete the assessment due to intellectual disability.

#### 2.2.2 Behavioural measures of face processing

**Fig. 1:**
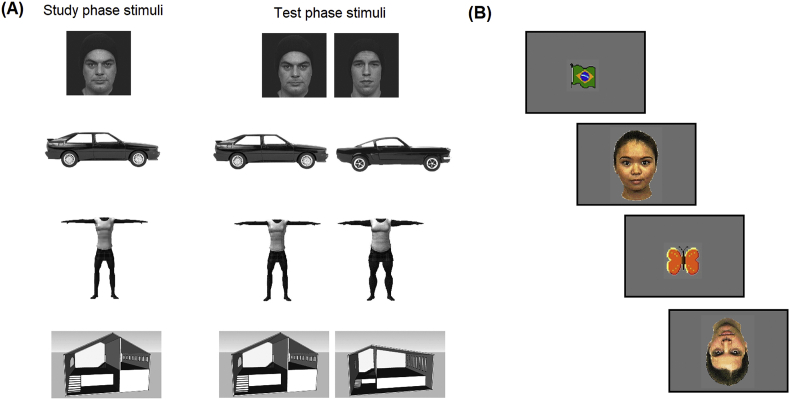
Stimuli used in the face recognition and face processing tasks in mid-childhood. Panel (A) shows examples of the face, car, body and scene stimuli used in the face recognition task in mid-childhood. Panel (B) shows examples of the upright and inverted face and fixation stimuli used in the EEG face processing task in mid-childhood.

Behavioural measures of face processing were derived from performance on a face recognition task. The face recognition task was a replication (with permission) of a paradigm developed by Weigelt et al. (2013) which revealed recognition memory impairments specific to social stimuli in 5-12 year-old children with ASD. The task (Fig. 1a) began with a study phase in which participants viewed 20 greyscale images presented individually for 3 sec in the centre of a 15-inch (1024 × 768 pixels) Lenovo ThinkPad laptop computer screen at a viewing distance of approximately 60 cm. Children were asked to look carefully at each image and try to remember it. There were two versions of the task: in the first version 10 face images (5.7° × 5.7° of visual angle) were shown followed by 10 car images (10.5° × 3.8°), and in the second version 10 body images (9.5° × 11.4°) were shown followed by 10 scene images (9.5° × 5.7°). Following Weigelt et al. (2013) stimuli were presented in a fixed order for all participants. Task versions were alternated in order across participants. A test phase immediately followed the study phase in which 10 pairs of face (or body) stimuli were presented followed by 10 pairs of car (or scene) stimuli. Each pair consisted of an “old” image (presented in the study phase) and a “new” image (not presented previously). Test phase stimuli were presented bilaterally on the left and right sides of the screen, with the old stimulus on the right 50% of the time. Participants indicated which image they saw during the study phase by pressing a left or right button (keyboard keys z and m, respectively, with keyboard language settings in English) corresponding to the side of the screen where the “old” image appeared. Each stimulus pair was shown for 10 sec or until a response was made, after which the next stimulus pair appeared on screen. The study and test phases were then repeated with the two image types the child had not seen in the first study and test phase (bodies and scenes for the first task version; faces and cars for the second task version). The task was programmed in E-Prime v2.0 (Psychology Software Tools Inc., 2012). Performance measures were recognition accuracy (% correct trials) and median reaction time for correctly recognised trials (RT, msec) per condition (faces, cars, bodies, scenes). Two HR children and four LR children did not complete the task due to time constraints (*n* = 5) or intellectual disability (defined as FSIQ < 69 on the WASI-II, *n* = 1). A further four HR and three LR children produced outlying performance measures (accuracy or RT 3SD ± group mean) and were also excluded, leaving 36 HR (12 boys, 24 girls) and 28 LR (13 boys, 15 girls) for analysis.

#### 2.2.3 Neurophysiological measures of face processing

Children were asked to complete a face processing task during EEG recording as part of a 1-h EEG task battery. Of the 42 HR and 35 LR children taking part in the mid-childhood visit, 21 HR children (7 boys, 14 girls) and 28 LR children (12 boys, 16 girls) completed the EEG face processing task (see Supplementary Materials for details of the EEG task battery, reasons for the remaining children not completing the face processing task and analysis examining potential biases between children who did and did not complete the EEG task).

The face processing task (Fig. 1b) was a passive viewing task in which participants were shown colour images (9.7° × 12.4° at a 60 cm viewing distance) of three female faces presented in upright or inverted orientation with direct gaze. Each task trial began with a fixation stimulus (a colour cartoon image, 3.8° × 3.8°) for a random 500–700 msec followed by an upright or inverted face image for 500 msec. The eye region appeared at the same location as the centre of the preceding fixation stimulus regardless of orientation. All stimuli were presented centrally on a 23” 16:9 monitor with a grey background. Four blocks of 42 trials were presented separated by short rest breaks, with 21 upright and 21 inverted faces presented in randomised order per block. Children were asked to look carefully at each face image. Attention to the screen was video-recorded throughout the task. Videos were coded offline and trials during which children were not watching the screen were excluded from analysis. The HR and LR groups did not differ significantly in the number of unattended trials (Table 2). The task was programmed in MATLAB R2013a (The Mathworks Inc., Natick, MA).

EEG was recorded from 62 Ag/AgCl active scalp electrodes placed according to the extended 10–20 system using an ActiCHamp DC-coupled recording system (Brain Products, Munich, Germany). The data were referenced online to electrode FCz and sampled at 500 Hz. EEG data were processed offline using Brain Vision Analyzer v2.03 (Brain Products, Munich, Germany). The data were filtered using .1 Hz high-pass, 40 Hz low-pass, 50 Hz notch Butterworth 24dB/Oct filters. Flat or noisy channels were removed and (if surrounded by four clean channels) interpolated using spherical spline interpolation. Independent Component Analysis (ICA) was used to identify and remove ocular artefact components. The data were segmented into −200 to 1000 msec stimulus-locked epochs surrounding the onset of face stimuli. Epochs with remaining artefacts, defined as those with amplitudes ± 90μv or a peak-to-peak amplitude change of 200μv, were rejected from further analysis. Finally, clean epochs were re-referenced to the average reference, baseline corrected using the −200 to 0 msec time-window and averaged to create ERPs for upright and inverted face conditions. Neurophysiological indices of face processing were peak amplitudes (mean ± 30 msec surrounding the peak amplitude) and latencies of the P1 and N170 ERP components. Based on parameters used previously (Tye et al., 2013) and inspection of grand and individual averages, the P1 was defined as the most positive peak within the 100–200 msec post-stimulus time-range at electrodes O1 (left hemisphere) and O2 (right hemisphere), and the N170 as the most negative peak within the 150–290 msec post-stimulus time-range at electrodes P7 (left hemisphere) and P8 (right hemisphere). Semi-automated peak-detection was used to identify peaks, i.e., maximum positive/negative peaks were automatically detected in the P1 and N170 time-windows and identified peaks were manually confirmed. The N170 was computed as a peak-to-peak measure with reference to the preceding positive peak (P1) at P7 and P8, respectively.

Two HR children were excluded from all ERP analysis due to having excessively noisy data and/or inattentiveness throughout the task. Three children were excluded from analysis of the P1 due to outlying amplitudes (+/− 3 SD outside of the group mean amplitude; HR *n* = 1) or excessive noise at the O1/O2 electrodes preventing measurement of the P1 (HR *n* = 1, LR *n* = 1). Two LR children produced outlying (3 SD ± group mean) peak-to-peak N170 amplitudes and were excluded from analysis of this component. Thus, analysis of the P1 was conducted with 17 HR and 27 LR children, and analysis of the N170 was conducted with 19 HR and 26 LR children. The HR and LR groups did not differ in the number of trials included in analysis in any condition (Table 2).

### 2.3 Assessments of face processing in infancy

#### 2.3.1 Neurophysiological correlates of face processing (Elsabbagh et al., 2012; Tye et al., In submission)

At the 7-month visit, infants completed a passive viewing task in which they were shown images of female faces and visual noise stimuli while their electrophysiological activity was recorded with a 128-channel HydroCel sensor net using a NetAmps 200 amplifier (Electrical Geodesics Inc., Oregon). Full details of the assessment are reported in Elsabbagh et al. (2012). For the purposes of the current study, amplitude and latency of the N290 ERP component, a negative-going component that is believed to be the infant precursor of the N170 component (Halit, De Haan, & Johnson, 2003), were computed for face and noise stimuli. Difference scores (face amplitude – noise amplitude; face latency – noise latency) were calculated to index face processing ability in infancy. Larger amplitude and latency difference scores (more negative for amplitude, more positive for latency) indicate larger (more negative) amplitude and longer (more positive) latency for face over noise stimuli; these patterns are associated with greater neural processing of faces (Halit et al., 2003). Group means for the N290 difference scores are shown in Table 2. Full details of data processing and computation of the N290 measures are reported in Elsabbagh et al. (2012) and Tye et al. (In submission). Previous analyses of these face/noise EEG data revealed atypicalities in N290 latency, i.e., a lack of differentiation in latency for faces versus noise, that were related to ASD outcomes at age 3 (Tye et al. In submission).

#### 2.3.2 Visual attention to face stimuli (Elsabbagh et al., 2013)

At age 7 months, infants completed a Face Pop-Out paradigm in which they were shown stimulus arrays consisting of one face image and four object images (e.g., car, bird, scrambled face, phone) while their eye-movement behaviour was recorded using a TOBII eye-tracker (for full details, see Elsabbagh et al., 2013). For the purposes of the current study, the proportion of time the infants spent looking at the face image compared to the object images was calculated and used in analysis to index attentional engagement with faces in infancy. Full details of data processing are provided in Elsabbagh et al. (2013). This measure of attentional engagement was previously found to be associated with poorer face recognition ability at age 3 years in this sample (de Klerk et al., 2014).

### 2.4 Statistical analysis (summarised in Table 1)

#### 2.4.1 Group differences in face processing in mid-childhood (Question/Hypothesis 1)

To test whether HR children would show poorer face recognition than LR children, accuracy and RT for each condition of the face recognition task (faces, cars, bodies, scenes) were entered into 2 (group: HR, LR) × 4 (condition) ANOVAs. Separate ANOVAs were used for accuracy and RT. Significant main effects of condition and condition × group interactions were further investigated using Bonferroni-corrected planned pairwise contrasts between the levels of each factor. To test whether HR children would show atypical neural processing of faces in mid-childhood, amplitudes and latencies of the P1 and N170 components from the EEG face processing task were entered into 2 (group) × 2 (orientation: upright, inverted) × 2 (hemisphere: left, right) ANOVAs. A separate model was used for amplitude and latency of each component. Significant interactions were further investigated using Bonferroni-corrected planned pairwise contrasts between the levels of each factor. All models were repeated including IQ and age as covariates, since these variables have been shown to influence face recognition and the P1 and N170 ERP components (Hileman et al., 2011; Kang et al., 2018, Luyster et al., 2019, Neuhaus et al., 2016). The results are reported wherever they differ from the main analyses.

Supplementary analyses were conducted to assess whether differences in face processing between the HR and LR groups were driven by the HR children with ASD and to investigate previously reported effects of sex on face processing atypicalities in HR children (Anzures et al., 2016). These analyses are described in full in the Supplementary Materials.

#### 2.4.2 Cross-sectional and longitudinal associations between face processing indices and ASD symptoms (Question/Hypothesis 2 and 3)

To examine how neural and behavioural indices of face processing relate to each other (Question/Hypothesis 2) and to ASD traits (Question/Hypothesis 3) in HR siblings in mid-childhood, Spearman correlation coefficients were computed between mid-childhood ERP (P1/N170) indices of face processing, face recognition ability (accuracy/RT for face trials), and mid-childhood ASD symptoms (SRS-2 and SSP scores). To limit the number of tests conducted, ERP indices reflecting typical face processing effects that were borne out in our data, i.e., inversion effects on P1 latency, with longer P1 latencies for inverted than upright faces, and lateralisation effects on N170 amplitude, with larger N170 amplitudes in the right than left hemisphere, were calculated [(Inverted P1 latency – Upright P1 latency)/(Inverted P1 latency + Upright P1 latency)]; [(right hemisphere N170 amplitude – left hemisphere N170 amplitude)/(right hemisphere N170 amplitude + left hemisphere N170 amplitude)] and used in correlations instead of raw latency/amplitude values. More positive values for these indices reflect greater right-lateralisation of the N170 and a larger inversion effect on P1 latency.

To examine whether face processing ability in infancy was associated with mid-childhood face processing ability and face recognition performance (Question/Hypothesis 2) and ASD symptoms (Question/Hypothesis 3) in HR siblings, Spearman correlation coefficients were computed between ERP indices of face processing in infancy (N290 amplitude/latency face *vs* noise difference scores) and mid-childhood ERP indices of face processing (P1 latency inversion effect, N170 amplitude lateralisation effect), mid-childhood face recognition performance (face trial accuracy/RT), and mid-childhood social-communication impairments and sensory symptoms.

Correlations were computed in the HR group since our goal was to understand face processing in children at risk for ASD and we expected HR children to show different associations between face processing variables and ASD traits compared to LR children (de Klerk et al., 2014). However, significant associations found in the HR group were examined in the LR group to test the latter assumption. We report correlation coefficients and their uncorrected *p*-values; in addition we highlight (with asterisks∗) the associations that remain significant with a Bonferroni correction applied for multiple testing (*p* = .05/12 = .004).

## 3 Results

### 3.1 Group differences in face processing in mid-childhood (Question/Hypothesis 1)

#### 3.1.1 Face recognition performance

**Fig. 2:**
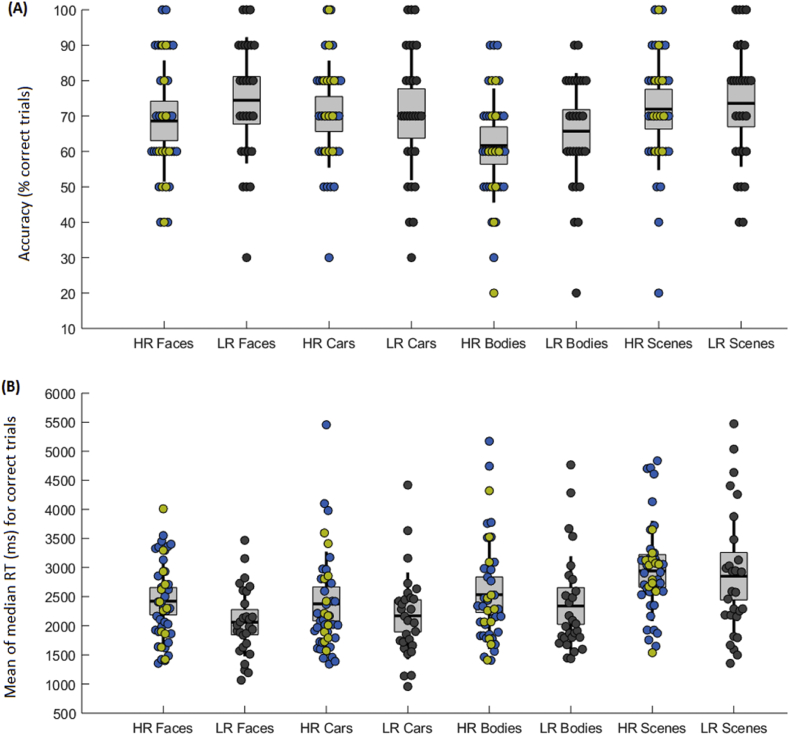
Performance in the face recognition task in mid-childhood. Boxplots display the group means (black line) and individual participants' scores (circles) for accuracy and RT performance in the face recognition task in mid-childhood. Panel (A) shows accuracy performance (% correct trials per condition) and Panel B shows the mean-of median RT for correctly recognised trials (ms). HR and LR group means and individual scores are presented in separate columns. Children in the LR group are indicated by grey circles; children in the HR group are indicated by blue circles, with the HR children who met diagnostic criteria for ASD highlighted in yellow.

Accuracy and RT performance are presented by group (HR, LR) and condition (faces, cars, bodies, scenes) in Fig. 2. There were significant main effects of condition on accuracy [*F*(3, 186) = 3.46, *p* = .02, *η*^*2*^ = .053] and RT [*F*(3, 186) = 17.44, *p* < .001, *η*^*2*^ = .220] performance; across groups, children were less accurate in recognising bodies than scenes (*p*^*Bonferroni*^ = .03, *d* = .55) and slower to correctly recognise scenes than faces, cars, or bodies (all *p*^*Bonferroni*^ < .001, *d* ≥ .52). There were no main effects of group and no group × condition interactions for accuracy or RT (all *F* ≤ 1.41, *p* ≥ .24, *η*^*2*^ ≤ .022). These results were unchanged when covarying age and IQ.

#### 3.1.2 Neurophysiological indices of face processing

**Fig. 3:**
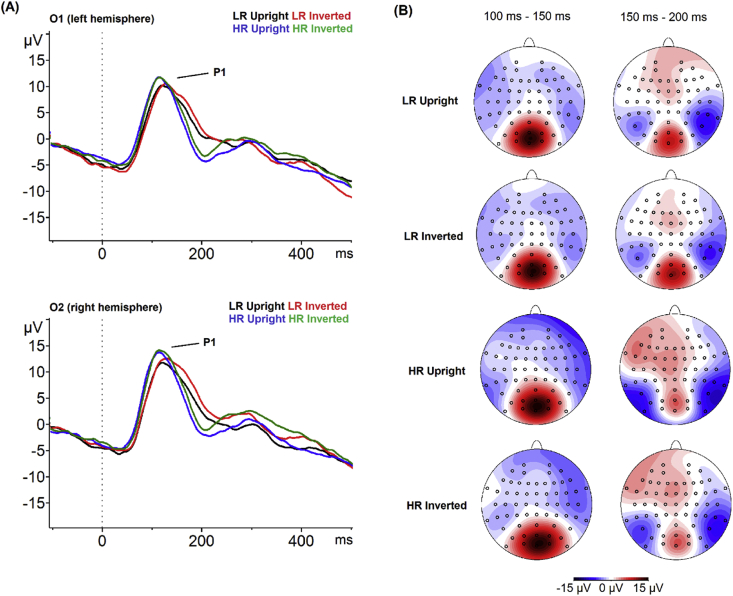
Grand average waveforms and topographical plots for the P1 component by group and condition. Panel (A) shows the grand average stimulus-locked waveforms displaying the P1 ERP component for upright and inverted faces by HR and LR group at electrode O1 (left hemisphere, top) and electrode O2 (right hemisphere, bottom). Black line = Grand average for the upright face condition in the LR group. Red line = Grand average for the inverted face condition in the LR group. Blue line = Grand average for the upright face condition in the HR group. Blue line = Grand average for the inverted face condition in the HR group. Panel (B) shows the topographical maps of the P1 component by group (LR, HR) and condition (upright and inverted faces).

*P1:* Grand averages for the P1 in HR and LR groups are shown in Fig. 3. For P1 amplitude, there were no main effects of group, orientation, or hemisphere and no interactions between these factors (all *F* ≤ 2.91, *p* ≥ .10, *η*^*2*^ ≤ .065). In contrast, for P1 latency, there were significant main effects of group [*F*(1, 42) = 5.48, *p* = .02, *η*^*2*^ = .115] and orientation [*F*(1, 42) = 5.59, *p* = .02, *η*^*2*^ = .117], reflecting significantly shorter P1 latencies in the HR than LR group across orientations and hemispheres and significantly shorter latencies in the upright than inverted condition across groups and hemispheres. There were no further significant main effects on P1 latency and no interactions between factors (all *F* ≤ 1.19, *p* ≥ .28, *η*^*2*^ ≤ .028). The significant main effect of group on P1 latency remained when controlling for age and IQ [*F*(1, 40) = 6.74, *p* = .01, *η*^*2*^ = .144]; all other effects were unchanged.

**Fig. 4:**
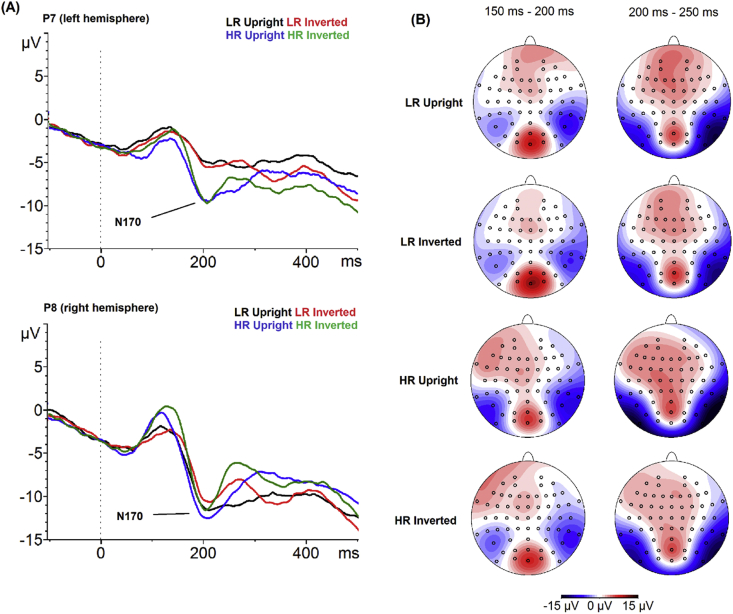
Grand average waveforms and topographical plots for the N170 component by group and condition. Panel (A) shows the grand average stimulus-locked waveforms displaying the N170 ERP component for upright and inverted faces by HR and LR group at electrode P7 (left hemisphere, top) and electrode P8 (right hemisphere, bottom). Black line = Grand average for the upright face condition in the LR group. Red line = Grand average for the inverted face condition in the LR group. Blue line = Grand average for the upright face condition in the HR group. Blue line = Grand average for the inverted face condition in the HR group. Panel (B) shows the topographical maps of the N170 component by group (LR, HR) and condition (upright and inverted faces).

*N170:* Grand averages for the N170 in HR and LR groups are shown in Fig. 4. For N170 amplitude, there were significant main effects of group [*F*(1, 43) = 6.64, *p* = .01, *η*^*2*^ = .134] and hemisphere [*F*(1, 43) = 34.96, *p* < .001, *η*^*2*^ = .448], reflecting significantly larger N170 amplitudes in the HR than LR group across orientations and hemispheres and significantly larger amplitudes in the right than left hemisphere across groups and orientations. There were no further significant main effects or interactions for N170 amplitude (all *F* ≤ 1.99, *p* ≥ .17, *η*^*2*^ ≤ .044). The significant main effect of group remained when controlling for age and IQ [*F*(1, 41) = 6.67, *p* = .01, *η*^*2*^ = .140]; all other effects were unchanged. For N170 latency, there were no main effects of group, orientation or hemisphere and no interactions between these factors (all *F* ≤ 1.56, *p* ≥ .22, *η*^*2*^ ≤ .035).

#### 3.1.3 Effects of ASD outcome and sex on face processing in mid-childhood

Effects of ASD outcome and sex on face recognition and face processing are reported in full in the Supplementary Materials. Briefly, the analysis of ASD outcome indicated that the HR children with ASD were not driving differences between the HR and LR groups in ERP indices of face processing, although it should be noted that the sample size for the HR-ASD group was very small for these measures (*n* = 5–6). For the face recognition task, sex interacted with group and condition for RT data, reflecting differences between the HR and LR groups (faster RTs in LR than HR for face and scene conditions) in boys but not in girls, and differences between boys and girls (faster RTs for faces, cars and scenes in boys than girls) in the LR group but not in the HR group. Sex did not influence face recognition accuracy or ERP indices of face processing.

### 3.2 Cross-sectional associations between face processing and ASD symptoms in mid-childhood (Question/Hypothesis 2 and 3)

**Fig. 5:**
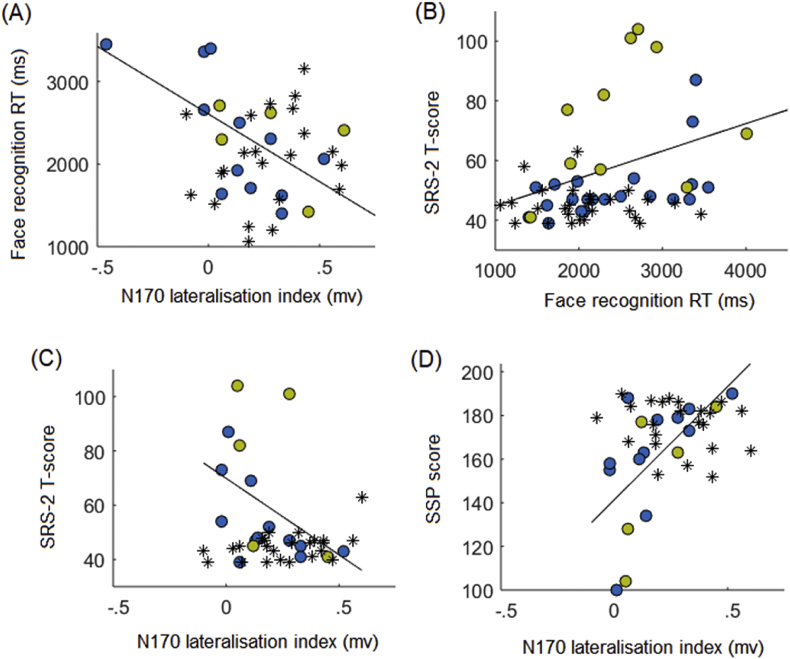
Cross-sectional associations between face recognition performance, face processing ERP indices and ASD symptoms in mid-childhood. Scatterplots show the associations between face recognition performance, ERP indices of face processing and ASD symptoms in mid-childhood in the HR group. The blue circles indicate data from the HR children without ASD and yellow circles indicate HR children with ASD; the regression lines represent the association between the variables in the HR group (HR-ASD and HR-non-ASD children combined). Black asterisks represent data points from the LR group and are shown only for visual comparison with the HR group associations. Panel (A) shows the negative association between RT for correctly recognising faces in the face recognition task and the extent to which the N170 ERP component for faces was lateralised to the right hemisphere; faster RTs were associated with greater right-lateralisation of the N170. Panel (B) shows the positive association between RTs for correctly recognising face stimuli in the face recognition task and SRS-2 scores; faster RTs were associated with fewer social-communication problems. Panel (C) shows the negative association between lateralisation of the N170 ERP component and SRS-2 scores; greater right-lateralisation of the N170 was associated with fewer social-communication problems. Panel (D) shows the positive association between lateralisation of the N170 ERP component and SSP scores; greater right-lateralisation of the N170 was associated with fewer sensory symptoms (higher SSP scores).

In the HR group, face recognition RT was significantly negatively correlated with the N170 lateralisation index [*rho*(17) = −.672, *p* = .003∗] and significantly positively correlated with SRS-2 scores [*rho*(31) = .467, *p* = .008], indicating that children who were faster to correctly recognise faces had a more right-lateralised N170 and fewer social-communication impairments (Fig. 5a–b). The N170 lateralisation index was significantly negatively correlated with SRS-2 scores [*rho*(17) = −.575, *p* = .02] and positively with SSP scores [*rho*(17) = .716, *p* = .001∗]; children with more right-lateralised N170 had fewer social-communication problems and sensory symptoms (Fig. 5c–d). A post-hoc analysis confirmed that the association between N170 lateralisation and sensory symptoms held for both hyper-sensitivity [*rho*(17) = .627, *p* = .007] and hypo-sensitivity [*rho*(17) = .662, *p* = .004∗] to sensory information (see Supplementary Materials). There were no further significant associations in the HR group (see Supplementary Materials for correlation matrix). These associations were not significant in the LR group (all *rho* ≤ .343, *p* ≥ .10; Fig. 5).

### 3.3 Longitudinal associations between infant face processing and mid-childhood face processing and ASD symptoms (Question/Hypothesis 2 and 3)

**Fig. 6:**
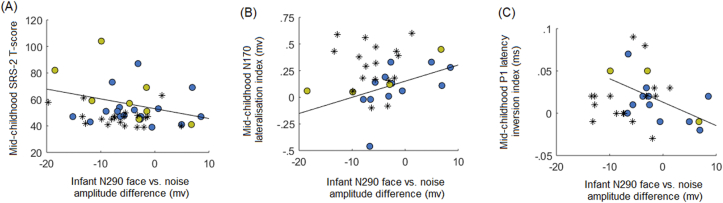
Longitudinal associations between face processing ERP indices in infancy and mid-childhood and mid-childhood ASD symptoms. Scatterplots show the associations between ERP indices of face processing at age 7-months and in mid-childhood and ASD symptoms in mid-childhood. The blue circles indicate data from the HR children without ASD and yellow circles indicate HR children with ASD; the regression lines represent the association between the variables in the HR group (HR-ASD and HR-non-ASD children combined). Black asterisks represent data points from the LR group and are shown only for visual comparison with the HR group associations. Panel (A) shows the negative association between the N290 amplitude difference score for face versus noise stimuli in infancy and SRS-2 scores in mid-childhood; larger (more negative) N290 difference scores (indicating larger N290 amplitude for face *vs* noise stimuli) were associated with more severe social-communication problems in mid-childhood. Panel (B) shows the positive association between the N290 amplitude difference score in infancy and the extent to which the N170 was right-lateralised in mid-childhood; larger (more negative) N290 difference scores (larger amplitudes for faces *vs* noise) were associated with less right-lateralisation of the N170 in mid-childhood. Panel (C) shows the negative association between the N290 difference score in infancy and the extent to which latency of the P1 was slower for inverted than upright faces in mid-childhood; larger (more negative) N290 difference scores (larger amplitudes for faces *vs* noise) were associated with larger latency increases for inverted versus upright faces in mid-childhood.

There were no significant longitudinal associations between attentional engagement during the face pop-out task in infancy and mid-childhood indices of face processing or social-communication or sensory symptoms in the HR group (all *rho* ≤ −.309, *p* ≤ .08, see Supplementary Materials for correlation matrix). However, the N290 difference score for faces versus noise in infancy was correlated positively with the mid-childhood N170 lateralisation index [*rho*(16) = .665, *p* = .005] and negatively with mid-childhood SRS-2 scores [*rho*(27) = −.425, *p* = .03] and P1 inversion effect [*rho*(14) = −.538, *p* = .047] in the HR group. This pattern indicates that HR infants with larger, more negative N290 amplitudes for faces versus noise (the more typical pattern indicating better face processing) showed less (more atypical) right-lateralisation of the N170, slower (more typical) P1 latency for inverted *vs* upright faces and higher social-communication problems in mid-childhood (Fig. 6a–c). To better understand these findings, a post-hoc analysis examined whether the associations between infant N290 and mid-childhood face processing and ASD symptoms held when examining N290 amplitudes for face and noise stimuli separately. This analysis indicated that these longitudinal associations were driven by HR infants’ processing of noise rather than face stimuli. The infant N290 amplitude for noise stimuli was significantly positively associated with mid-childhood SRS-2 scores [*rho*(29) = .560, *p* = .002∗] and the P1 lateralisation index [*rho*(14) = .609, *p* = .02] and significantly negatively correlated with the mid-childhood N170 lateralisation index [*rho*(16) = −.629, *p* = .009]. In contrast, the infant N290 amplitude for face stimuli was not associated with mid-childhood SRS-2, N170 or P1 measures (all *rho* ≤ −.238, *p* ≥ .37, see Supplementary Materials for full results and scatterplots). The associations between the infant N290 and mid-childhood face processing and social-communication symptoms were non-significant in the LR group (all *rho* ≤ .309, *p* ≥ .21; Fig. 6).

## 4 Discussion

### 4.1 Face processing in high-risk siblings in mid-childhood

In contrast to our predictions (Question/Hypothesis 1, see Table 1), our main analysis revealed no differences between the high-risk and low-risk groups in performance on the face recognition task in mid-childhood. There were also no differences related to ASD outcomes. However, our supplementary analysis modelling sex showed that high-risk boys were significantly slower to recognise face and scene stimuli than low-risk boys, while high-risk girls were comparable to low-risk girls. These findings indicate that boys with familial risk for ASD had difficulty recognising faces and other complex non-face stimuli. Anzures et al. (2016) similarly reported atypicalities in face and object processing that were restricted to high-risk boys, although the atypicalities were found for neural correlates and not, as in our study, for recognition performance. Still, overall this pattern of findings might indicate that high-risk girls are better able to compensate for difficulties with face and object processing associated with familial risk for ASD and/or that boys express more of these risk characteristics than do girls, a phenomenon termed the *female protective effect* (Robinson, Lichtenstein, Anckarsäter, Happé, & Ronald, 2013). Future work in larger longitudinal samples could test this interpretation by examining whether face processing in high-risk boys is more affected by earlier face processing atypicalities than it is in high-risk girls. In the current study, our sample size was too small to investigate longitudinal associations in high-risk boys and girls separately.

In terms of the neural correlates of face processing in mid-childhood, both high-risk and low-risk siblings showed typical effects of face inversion on P1 latency (longer latency to inverted than upright faces) and larger N170 amplitude in the right than left hemisphere. The inversion effect on P1 latency is believed to reflect increased or prolonged attention to the more-difficult-to-identify inverted face, while the right-lateralisation of the N170 is believed to reflect greater activity of right-hemisphere brain regions specialised for face-selective processes, including extraction of configural information and face categorisation (Taylor, Batty, & Itier, 2004). The presence of these characteristics suggests typical development of these aspects of face processing in both high-risk and low-risk groups. Nevertheless, the high-risk siblings showed significantly shorter P1 latencies and significantly larger N170 amplitudes across hemispheres and conditions than the low-risk group, indicative of subtle atypicalities in neural correlates of face processing. One interpretation of these findings is that the high-risk group showed reduced early attentional processing of faces (shorter P1 latencies) but subsequently engaged neural circuitry involved in later stages of face processing to a greater degree (enhanced N170 amplitudes) than low-risk children. Indeed, enhanced N170 amplitudes were reported in one previous study (Anzures et al., 2016) in high-risk siblings in mid-childhood and were interpreted as reflecting greater recruitment of neural resources for efficient visual processing, although the effect was also found for non-face object stimuli and was only present in high-risk boys and not girls. An alternative interpretation is that the high-risk group showed superior face processing abilities, since shorter P1 latencies and larger N170 amplitudes are typically found in older individuals and in those without ASD (Hileman et al., 2011; Luyster et al., 2019, Kovarski et al., 2019, Neuhaus et al., 2016); that the subset of high-risk siblings with ASD did not show shorter P1 latencies and increased N170 amplitudes might support this suggestion, although the sample size of the HR-ASD group is too small (*n* = 5) to draw firm conclusions. Furthermore, the HR group did not show better face recognition performance than the LR group, which might be expected if neural processing of faces was superior in the HR children.

In addition to these group differences, our dimensional analysis revealed several associations between face recognition performance, neural correlates of face processing and ASD symptoms in the high-risk group that were consistent with our hypotheses (Question/Hypothesis 2 and 3, see Table 1). High-risk children with the slowest RTs to recognise face stimuli and the least right-lateralised N170 showed the most severe social-communication impairments. Furthermore, behavioural and neural indices of face processing were correlated with each other: high-risk children who were slowest to recognise faces also had the least right-lateralisation of the N170. This pattern of findings highlights potentially important links between the integrity of right-lateralised face processing circuitry, children's ability to recognise faces and their everyday-life social abilities and is consistent with the proposal that there is a lack of specialisation in social brain networks in ASD (Webb et al., 2017), a hypothesis derived from the interactive specialisation model of the typical development of face processing (Johnson, 2011).

Finally, less right-lateralisation of the N170 was also associated with more severe sensory symptoms and this association remained significant when examining sensory hypo-responsiveness and sensory hyper-sensitivity separately. These novel findings appear to contradict previous work in toddlers with ASD and high-risk infants showing that greater sensory hyper-sensitivity predicts more typical neural correlates of face processing and better social abilities, a finding that was interpreted to reflect facilitatory effects of increased salience and attentional capture by social stimuli as a result of hyper-sensitivity to incoming sensory information (Jones et al., 2018, see also; Green, Ben-Sasson, Soto, & Carter, 2012). In contrast, our findings suggest that hypo- and hyper- sensory sensitivity interferes with successful face processing. Still, it should be noted that Jones et al.’s (2018) associations were between hyper-sensitivity and the P1 ERP rather than the N170 (or the infant equivalent – N290) and were only present longitudinally (with earlier hyper-sensitivity predicting higher P1 amplitudes later in life) and were not significant between concurrent measures of sensory symptoms and face processing correlates as in our study. Similar to our findings, a recent study in adolescents with ASD showed that sensory stimulation during a social cognition task was associated with reduced activity in temporal and prefrontal regions required for successful task performance and increased activity in sensory processing regions, and that greater activity in the latter regions were also associated with greater sensory over-responsivity (Green, Hernandez, Bowman, Bookheimer, & Dapretto, 2018, see also; Hilton, Graver, & LaVesser, 2007). One possible explanation for these discrepant findings is that hyper-sensitivity may be helpful in enhancing attention towards social stimuli early in life, i.e., in infancy and toddlerhood (Green et al., 2012, Jones et al., 2018) but becomes interfering as development progresses and cognition becomes more complex (Green et al., 2018, Hilton et al., 2007). Alternatively, the extent to which sensory symptoms enhance or interfere with face processing may vary across individuals with or at risk for ASD.

### 4.2 Longitudinal associations between face processing in infancy and mid-childhood face processing and ASD symptoms

To our knowledge, we are the first to report on how face processing in the first year of life associates with face processing abilities and ASD symptoms in mid-childhood in high-risk siblings. In contrast to our predictions (Question/Hypothesis 2 and 3, see Table 1), our findings showed that high-risk infants with the most typical neural correlates of face processing, i.e., greater enhancement of the N290 component for face versus noise stimuli, at age 7 months had more severe social-communication impairments, less right-lateralisation of the N170 and more typical face inversion effects on P1 latencies (longer latencies for inverted than upright faces) in mid-childhood. Further investigation showed that these associations were driven by high-risk infants’ processing of non-face visual noise stimuli rather than face stimuli: high-risk infants with smaller N290 amplitudes for noise stimuli (which would contribute to a larger N290 difference score for faces *vs* noise) had higher social-communication problems and less right-lateralised N170 components but longer P1 latencies for inverted than upright faces later in childhood. In contrast, there were no associations between the N290 amplitude for faces in infancy and mid-childhood face processing or social-communication measures. These findings might suggest that high-risk infants with less efficient processing of non-face object stimuli in the first year of life (indicated by smaller N290 amplitudes for noise) show less specialised neural processing of faces (less N170 lateralisation), slowed processing of object-like inverted face stimuli (slower P1 latency for inverted faces) and greater social-communication difficulties later in childhood. Alternatively, the smaller N290 amplitudes in infancy might reflect more efficient neural processing of noise stimuli (requiring fewer neurocognitive resources) in which case the pattern of findings would indicate that high-risk infants with better neurocognitive processing of non-face object stimuli show less specialised face processing and greater social-communication symptoms later in childhood. This latter interpretation is consistent with two previous studies that reported atypicalities in neural correlates of object processing in high-risk infants (McCleery et al., 2009) and in toddlers with ASD (Webb, Dawson, Bernier, & Panagiotides, 2006). The pattern of findings from those studies indicated that high-risk infants and young children with ASD showed enhanced object over face processing, leading to the proposal that the early development of ASD may be associated with preferential processing of non-social stimuli such as objects at the expense of processing social stimuli such as faces, resulting in atypical development of face processing (McCleery et al., 2009, Webb et al., 2006). Together these findings indicate that non-face object processing may be disrupted in the early development of ASD and highlight that existing perceptual/cognitive models, which propose that early disruptions to perceptual, cognitive and neural systems underlying face processing impede typical development of this ability and increase risk for social impairments in individuals with or at risk for ASD (Dawson et al., 2005b, Schultz, 2005), are likely not sufficient to account for the development of social-communication problems in ASD. The N290 index was not associated with mid-childhood sensory symptoms or behavioural face recognition performance in high-risk children. These findings suggest that early alterations in neural correlates of object processing might specifically influence the severity of later social-communication problems and not sensory processing atypicalities, and that mid-childhood face recognition problems may arise as a consequence of concurrent neural processing problems but not directly from such problems in infancy (or at least, not from the face processing indices that we measured in infancy).

Finally, there were no significant associations between visual attention to face stimuli in infancy and mid-childhood face processing abilities or ASD symptoms. These findings contradict social attention models, which propose that reduced attention to faces and other social stimuli in infancy impairs the neurocognitive development of face processing and leads to deficits in social-communication abilities that rely, in part, on the efficient use and integration of information acquired from faces (Dawson et al., 2005b, Webb et al., 2017). Still, it should be noted that our sample of high-risk siblings showed increased rather than decreased attention to faces in infancy (Elsabbagh et al., 2013) unlike other samples of high-risk infants who later meet diagnostic criteria for ASD (Chawarska et al., 2013, Jones et al., 2016). It is possible that visual attention to face stimuli in infancy does contribute to later childhood face processing and social-communication abilities amongst infants who do show reduced attention to faces. Furthermore, increased attention to face stimuli in our sample of high-risk infants was associated with poorer face recognition ability earlier in life, at age 3 years (de Klerk et al., 2014). Atypicalities in social attention in infancy may therefore influence the early development of face processing in high-risk children, but do not relate directly to measures of face processing ability assessed later in childhood. A final consideration is that early eye-tracking measures of attention to face stimuli may index cognitive processes other than social attention. A recent study with high-risk infants showed that longer look durations to face stimuli in the Face Pop-out task were negatively associated with executive functions at age 3 years and were not associated with the severity of social-communication impairments (Hendry et al., 2018). Future work modelling pathways between early social attention measures and later executive functions, face processing abilities and social-communication skills will be important to clarify the role that visual attention to faces in infancy plays in the development of ASD.

### 4.3 Limitations

The face recognition task we used did not yield typical ‘face advantage’ effects, i.e., higher accuracy and faster RTs for the face compared to non-face conditions. While this task has previously been used in a large (*n* = 100) sample of children of a similar age to our sample and revealed memory impairments specific to social stimuli (faces and bodies) in children with ASD (Weigelt et al., 2013), it may have been better to use a more established task which robustly yields face advantage effects (e.g., the Cambridge Face Memory Test; Duchaine & Nakayama, 2006). We did not include a non-face object control condition in our EEG face processing task and consequently we cannot rule out the possibility that altered neurophysiological correlates in the high-risk group were not specific to face processing. We did not find effects of face inversion on amplitude or latency of the N170, which might suggest that face processing was in some way atypical in our sample since these effects are frequently reported in the literature (Bentin et al., 1996, Ince et al., 2016, Itier and Taylor, 2004). We note, however, that some cross-sectional studies have found N170 inversion effects to be absent in young (aged <10–11 years) typically developing children and only present in older (>12 years) children and adolescents (Taylor et al., 2004). Thus, the absence of face inversion effects on the N170 in our study may reflect the young age (6–8 years) of our participants.

There was considerable drop-out from the EEG task in mid-childhood and while the children who did and did not complete the task did not differ on age, sex, IQ, ASD symptom severity, face recognition performance in mid-childhood or visual attentional engagement with faces in infancy, the children who did not complete the task had a significantly more negative N290 amplitude difference score for faces versus noise in infancy than the children who did complete the task (see Supplementary Materials for analysis results). Since a more negative N290 amplitude difference score is indicative of larger enhancement in the N290 for faces versus noise, it is possible that the children included in analysis of mid-childhood face processing ERPs in the current study had somewhat poorer face processing abilities, at least in terms of the N290 infant neural marker of face processing, than those who dropped out of the task. A more powerful statistical analysis approach than the correlational analysis we conducted here would have been to use structural equation models or path analysis to model developmental pathways in infant to mid-childhood measures of face processing and ASD symptoms. This approach was not possible in the current study due to our modest sample size. Most children included in our analysis had IQs within the average (85–115) range and future studies should investigate the development of face processing in children with or at-risk for ASD and a wider range of intellectual abilities.

## 5 Conclusions

Compared to siblings at low familial risk for ASD, high-risk siblings showed atypical P1 and N170 neurophysiological correlates of face processing in mid-childhood and, for high-risk boys only, poorer face recognition performance. Within the high-risk group, less right-lateralisation of the N170 was associated with poorer face recognition performance and higher social-communication and sensory symptoms. Interestingly, more atypical face processing, i.e., less right-lateralised N170 amplitudes, and higher social-communication problems in mid-childhood associated with atypical neural correlates of object (but not face) processing in infancy. These findings indicate that face processing ability, particularly the function of neural circuitry specialised for faces, may play an important role in the development or maintenance of ASD symptoms and that disruptions to object processing in the first year of life appear to influence later face processing and social functioning.

## Data statement

PDF files containing the SPSS syntax and statistical analysis outputs as well as summary statistics for all analyses are available to download from the public OSF data repository here: https://osf.io/jzx75/. Our ethical approval for this study does not include permission to upload data files to a public data repository and parents of children participating in the study only consented to their child's data being shared with scientists outside of the research team if those scientists were working collaboratively with the BASIS Network (for further details see the BASIS network website: http://www.basisnetwork.org/collaboration-and-project-affiliation/). Thus, scientists and others interested in accessing the data files should complete a project affiliation form (available here: http://www.basisnetwork.org/collaboration-and-project-affiliation/), stating their reason(s) and aim(s) for becoming affiliated with the network and send this to the BASIS network administrators (basis@bbk.ac.uk). Proposed project affiliations are reviewed by scientific board members of the BASIS network based on the criteria described here (http://www.basisnetwork.org/collaboration-and-project-affiliation/). The experimental tasks used in this study (upright and inverted face processing and face recognition tasks) were developed by scientists outside of the study team and were used in this study after seeking permission from those scientists. For this reason, we have not uploaded the programme files and stimuli for the experiments to the OSF repository; individuals wishing to access the stimuli and experimental programme files should request these from the original authors (email Leslie Tucker at the Centre for Brain and Cognitive Development, l.tucker@bbk.ac.uk, to access the face processing task and Dr. Kami Koldewyn at Bangor University, k.koldewyn@bangor.ac.uk, to access the face recognition task). The procedures and analysis methods for this study were not pre-registered. We report how we determined our sample size, all data exclusions, all inclusion/exclusion criteria, whether inclusion/exclusion criteria were established prior to data analysis, all manipulations, and all measures in the study.

## CRediT author statement

ES collected and processed the mid-childhood data (Project administration, Data curation), designed and conducted the analysis (Conceptualization, Formal analysis, Methodology) and drafted the manuscript (Roles/Writing – original draft). BM collected and processed the data (Project administration, Data curation) and helped to draft the manuscript (Writing – review and editing). LM designed and programmed the EEG tasks and EEG processing and analysis methods (Conceptualization, Formal analysis, Methodology) and helped to draft the manuscript (Writing – review and editing). ME designed the methods and tasks, collected and processed data and designed and conducted analysis for the infant phases of the study (Project administration, Data curation, Conceptualization, Formal analysis, Methodology) and helped to draft the manuscript (Writing – review and editing). CT processed data and conducted analysis of the infant EEG data (Data curation, Formal analysis) and helped to draft the manuscript (Writing – review and editing). TG helped to design the study (Conceptualization, Methodology) and draft the manuscript (Writing – review and editing). EJH helped to design the study (Conceptualization, Methodology), advised on design and execution of the analysis (Methodology, Supervision) and helped to draft the manuscript (Writing – review and editing). TC conceived of the study (Conceptualization, Methodology), supervised execution of the study (Supervision), advised on design and execution of the analysis (Methodology, Supervision) and helped to draft the manuscript (Writing – review and editing). MJH conceived of the study (Conceptualization, Methodology), supervised execution of the study (Supervision), advised on design and execution of the analysis (Methodology, Supervision) and helped to draft the manuscript (Writing – review and editing).

## Declaration of Competing Interest

The authors have no conflicts of interests.

## Footnotes

2 5 DAWBA and 5 SCQ missing.
