# Supplementary material for: Neural and behavioural indices of face processing in siblings of children with autism spectrum disorder (ASD): A longitudinal study from infancy to mid-childhood
Source: Cortex. 2020 Jun;127:162–79. doi: 10.1016/j.cortex.2020.02.008 (PMC7254063; doi:10.1016/j.cortex.2020.02.008)
Supplement: Multimedia component 1 [file mmc1.docx]

Supplementary materials

*Participants: assessment of retention biases*

Of the 104 infants (54 HR and 50 LR) initially recruited into the study in infancy, 53 HR (98%) and 48 LR (96%) children were retained up to age 3 years – the first time-point at which ASD outcomes were assigned and the last assessment time-point before the mid-childhood (age 7 years) follow-up study. Of the children retained to age 3 years, 44 HR (83%) and 37 LR (77%) were retained at the mid-childhood follow-up. The proportion of children retained between age 3 years and age 7 years did not differ between HR and LR groups (χ^2^ (1) = .844, *p* = .36). Possible retention biases were investigated by comparing symptoms of ASD, adaptive behaviour and developmental ability measured at age 3 years between the children retained to age 7 years and those not retained to age 7 years in the HR and LR groups separately. Age 3 ASD symptoms were assessed using the *Autism Diagnostic Observation Schedule – Second Edition* (*ADOS-2*; Lord et al., 2012), the *Social Communication Questionnaire (SCQ;* Rutter et al., 2003), and the *Social Responsiveness Scale – Second Edition* (*SRS-2*; Constantino, 2012); age 3 adaptive behaviour was assessed using the *Vineland Adaptive Behavior Scales – Second Edition, Vineland-II*; Sparrow et al., 2005) and developmental ability using the *Mullen Scales of Early Learning* (*MSEL;* Mullen, 1995). In the HR group, the retained children did not differ from the non-retained children in 3-year levels of ASD symptoms (all *p* > .52), adaptive functioning (*p* = .39), or developmental ability (*p* = .41). Similarly, the retained LR children did not differ from the non-retained LR children in 3-year ASD traits (all *p* > .85), adaptive functioning (*p* = .55) or developmental ability (*p* = .31).

*Assessment of ASD symptoms and assignment of research diagnoses of ASD in mid-childhood*

The *Autism Diagnostic Observation Schedule – Second Edition* (*ADOS-2*; Lord et al., 2012), a standardised interaction observation assessment, was used to assess current symptoms of ASD (module 3 for 73 children, module 2 for one child, module 1 for one child; ADOS not completed with 1 LR child). The *Autism Diagnostic Interview – Revised* (*ADI-R*; Le Couteur et al., 2003), a structured parent interview, was completed with parents of HR children. Parents completed questionnaires to obtain further information on ASD symptoms and subclinical ASD traits. The *Social Responsiveness Scale – Second Edition* (*SRS-2*; Constantino, 2012) assessed parent-rated social impairments over the past 6 months. The *Short Sensory Profile* (*SSP*; Dunn, 1999) assessed parent-rated sensory processing difficulties. Higher scores on the SRS-2 reflect more severe ASD symptoms; lower scores on the SSP reflect greater sensory processing problems.

Experienced researchers who conducted the assessments (ES, BM, GP) and the lead clinician (TC) reviewed information on ASD symptomatology (ADOS-2, ADI-R (HR only), SCQ), adaptive functioning (measured by the *Vineland Adaptive Behavior Scales – Second Edition, Vineland-II*; Sparrow et al., 2005), and IQ (measured by the *Wechsler Abbreviated Scale of Intelligence – Second Edition*, *WASI-II*; Wechsler, 2011) for each HR and LR child and as a team assigned clinical consensus best estimate diagnosis of ASD according to DSM-5 (American Psychiatric Association, 2013). Of 42 high-risk children with complete ASD assessments in mid-childhood, 15 (7 boys, 8 girls) met DSM-5 (APA, 2013) criteria for ASD and the remaining 27 children (8 boys, 19 girls) did not. None of the 35 low-risk children met DSM-5 criteria for ASD and none had a community clinical ASD diagnosis.

*Mid-childhood EEG task battery and participant exclusions*

All children were asked to complete a one-hour task battery during EEG recording, comprised of (in order of presentation): 1) an eyes-open and eyes-closed resting-state recording, 2) an auditory oddball task, 3) a face processing task, and 4) a passive viewing task. Of 42 HR and 35 LR children taking part in the mid-childhood follow-up visit, 29 HR and 32 LR children completed all or part of the EEG battery. Reasons for the remaining 13 HR children not performing the EEG battery included: intellectual or behavioural problems that interfered with assessments (*n* = 4), time constraints (*n* = 2), being unable to tolerate the gel and cap (*n* = 5), and laboratory conditions (excessive heat) affecting electrode impedances (*n* = 2). Reasons for the 3 LR children not performing the EEG battery were time constraints (*n* = 2) and being unable to tolerate the cap/gel (*n* = 1). A further 8 HR children and 4 LR children did not complete the face processing task due to laboratory conditions (excessive heat, *n* = 5), fatigue by this point in the task battery (*n* = 4), or technical problems with the recording (*n* = 3). Thus, EEG data from the face processing task were available from 21 HR (7 boys, 14 girls) and 28 LR (12 boys, 16 girls) children for analysis. The sample of children who completed the face processing task did not differ in age, IQ, SRS-2 or SSP scores, face recognition performance in mid-childhood or visual attentional engagement with faces in infancy from those who did not complete the task (all *t* < -1.57, all *p* > .12). The groups also did not differ in sex or ASD outcome in mid-childhood (both χ^2^ < .208, *p* > .75). However, the children who did not complete the EEG task in mid-childhood showed a significantly more negative N290 amplitude difference score for faces vs. noise in infancy than those who did complete the EEG face task in mid-childhood (-8.67μv vs. -4.92μv; *t*(54) = 2.02, *p* = .048). Since a more negative N290 amplitude difference score reflects greater enhancement of the N290 for face vs. noise stimuli, this finding might indicate that the children included in the analysis of neural correlates of face processing in mid-childhood had somewhat poorer face processing abilities than those who did not.

*Effects of ASD outcome on face processing in mid-childhood*

To assess whether atypicalities in face recognition or neural correlates of face processing in the HR group were driven by children with ASD, we conducted a supplementary analysis in which we compared face recognition performance and ERP indices of face processing between HR children who met diagnostic criteria for ASD at age 7 (HR-ASD group), HR children who did not meet criteria for ASD at age 7 (HR-non-ASD group) and LR children. Two of the 27 HR children who did not meet diagnostic criteria for ASD in mid-childhood had met diagnostic criteria for ASD at age 3 years (reported in Shephard et al., 2017) and were excluded from analysis of ASD outcome effects on face processing. The number of children in each group included in analyses is shown in Table S1. The effects of ASD outcome on face processing were investigated using 3 (group: HR-ASD, HR-non-ASD, LR) x 4 (condition: faces, cars, bodies, scenes) ANOVAs for face recognition task accuracy and RT performance, and 3 (group: HR-ASD, HR-non-ASD, LR) x 2 (orientation: upright, inverted) x 2 (hemisphere) ANOVAs for amplitudes and latencies of the P1 and N170 ERP components. Significant main effects and interactions were further investigated using Bonferroni-corrected planned pairwise contrasts. We note that the size of the HR-ASD group for the ERP indices of face processing is small (*n* = 5) and highlight that this analysis is under-powered and should be considered preliminary.

*Face recognition:* accuracy and RT performance are presented by ASD outcome group and condition in Table S1. The 3 x 4 ANOVA on accuracy revealed a main effect of condition (*F*(3, 174) = 3.87, *p* = .01, *η^2^* = .063), which reflected lower accuracy for bodies than cars or scenes (all *p* < .04, *d* > .44), but no main effect of ASD outcome group or interaction between ASD outcome and condition (both *F* < .920, *p* > .40, *η^2^* < .031). Similarly, the 3 x 4 ANOVA on RT revealed a significant main effect of condition (*F*(3, 174) = 12.29, *p* < .001, *η^2^* = .175), reflecting longer RTs for scenes than all other stimuli (all *p* < .001, *d* > .46), but no main effect of ASD outcome group or interaction between these factors (both *F* < .683, *p* > .51, *η^2^* < .023).

*Face processing ERPs:* P1 and N170 amplitudes and latencies are presented by ASD outcome group in Table S1. There was a significant main effect of ASD outcome group on P1 latency (*F*(2, 40) = 4.01, *p* = .03, *η^2^* = .167), which reflected shorter P1 latencies in the HR-non-ASD group than in the LR group (*p* = .02, *d* = .82). The HR-ASD group did not differ significantly from the HR-non-ASD or LR groups on P1 latency (both *p* > .60, *d* < .68). There was also a significant main effect of orientation with shorter P1 latencies in the upright than inverted condition (*F*(1, 40) = 4.81, *p* = .03, *η^2^* = .107). There was a significant main effect of ASD outcome group on N170 amplitude (*F*(2, 41) = 3.74, *p* = .03, *η^2^* = .154), which reflected significantly larger N170 amplitudes in the HR-non-ASD group than in the LR group (*p* = .03, *d* = .75). The HR-ASD group did not differ significantly from the HR-non-ASD or LR groups (both *p* > .61, *d* < .47). There was also a significant effect of hemisphere (*F*(1, 41) = 22.15, *p* < .001, *η^2^* = .351), reflecting that N170 amplitude was larger in the right than left hemisphere across groups and orientations. There were no significant main effects or interactions for P1 amplitude or N170 latency (all *F* < 2.02, *p* > .16, *η^2^* < .048).

**Table S1** Means (SDs) for mid-childhood face recognition performance and face processing ERP indices presented by ASD outcome group

|  | **HR-ASD** | **HR-non-ASD** | **LR** |
| --- | --- | --- | --- |
| ***Face recognition task*** |  |  |  |
| *N* participants | 12 | 21 | 28 |
| Face accuracy | 65.00 (15.67) | 67.62 (17.01) | 72.07 (19.34) |
| Car accuracy | 75.00 (12.43) | 68.57 (16.52) | 70.34 (18.61) |
| Body accuracy | 58.33 (16.97) | 63.33 (16.53) | 65.52 (16.17) |
| Scene accuracy | 74.17 (12.40) | 70.48 (20.37) | 72.41 (18.64) |
| Face RT | 2445.33 (732.89) | 2399.98 (727.67) | 2060.43 (574.13) |
| Car RT | 2371.54 (661.69) | 2365.67 (975.05) | 2180.83 (733.09) |
| Body RT | 2456.54 (828.55) | 2597.07 (1033.49) | 2344.81 (838.36) |
| Scene RT | 2887.58 (514.49) | 2931.48 (1036.49) | 3020.31 (1418.33) |
| ***Face processing EEG task*** |  |  |  |
| *N* participants (P1, N170) | 5, 6 | 11, 12 | 27, 26 |
| Upright P1 latency O1 | 170.76 (13.52) | 159.47 (15.75) | 169.74 (18.14) |
| Upright P1 amplitude O1 | 16.41 (11.47) | 12.42 (4.86) | 11.76 (9.80) |
| Upright P1 latency O2 | 159.08 (9.40) | 153.96 (15.01) | 171.60 (19.86) |
| Upright P1 amplitude O2 | 17.27 (6.72) | 15.22 (8.92) | 13.51 (9.44) |
| Inverted P1 latency O1 | 174.00 (14.46) | 160.89 (15.65) | 176.45 (14.38) |
| Inverted P1 amplitude O1 | 14.67 (11.60) | 11.82 (6.28) | 11.91 (8.76) |
| Inverted P1 latency O2 | 171.12 (18.63) | 159.67 (17.53) | 175.07 (26.45) |
| Inverted P1 amplitude O2 | 15.83 (8.19) | 16.00 (10.13) | 14.50 (9.89) |
| Upright N170 latency P7 | 249.30 (50.51) | 240.55 (46.43) | 255.94 (37.64) |
| Upright N170 amplitude P7 | -9.76 (6.25) | -12.38 (4.82) | -7.28 (2.32) |
| Upright N170 latency P8 | 257.47 (14.03) | 250.90 (26.33) | 263.29 (26.96) |
| Upright N170 amplitude P8 | -16.64 (5.78) | -15.69 (6.19) | -13.89 (6.99) |
| Inverted N170 latency P7 | 267.63 (11.43) | 232.78 (39.05) | 250.45 (39.41) |
| Inverted N170 amplitude P7 | -10.75 (6.06) | -13.28 (4.77) | -7.54 (2.42) |
| Inverted N170 latency P8 | 257.00 (11.55) | 249.42 (19.90) | 252.18 (32.92) |
| Inverted N170 amplitude P8 | -14.59 (5.18) | -16.05 (6.48) | -13.34 (6.33) |

*Face recognition task accuracy* = % correct trials for recognising face, car, body and scene stimuli. *Face recognition task RT* = mean of median RT (ms) for correctly recognised trials per condition. *Upright* = upright face trials. *Inverted* = inverted face trials. *O1/O2/P7/P8* = electrode locations in the left (O1/P7) and right (O2/P8) hemispheres. *Amplitude* = peak amplitude (µv) of the P1 component and the peak-to-peak amplitude (P1-N170) of the N170 component. *Latency* = latency (ms) of the peak P1 and N170 components.

*Effects of sex on face processing in mid-childhood*

One previous study examining event-related potential (ERP) correlates of face and object processing in 6-9-year-old high-risk siblings reported enhanced amplitudes of the P1 and N170 ERP components to face and house stimuli in high-risk boys compared to low-risk boys, but no difference in these ERP indices between high-risk and low-risk girls (Anzures et al., 2016). We conducted a supplementary analysis to assess whether similar effects of sex on face processing were present in our data. Effects of sex on face recognition performance and electrophysiological indices of face processing were investigated using 2 (group: HR, LR) x 2 (sex) x 4 (condition: faces, cars, bodies, scenes) ANOVAs for face recognition task accuracy and RT, and 2 (group: HR, LR) x 2 (sex) x 2 (orientation: upright, inverted) x 2 (hemisphere) ANOVAs for amplitudes and latencies of the P1 and N170 ERP components. Significant main effects and interactions were further investigated using Bonferroni-corrected planned pairwise contrasts. Effects of sex and interactions between sex and other factors are reported below; effects of group, condition, orientation and hemisphere are only reported if they differ from the results reported in the main text.

*Face recognition:* accuracy and RT performance are presented by group, sex, and condition in Table S2. The 2 x 2 x 4 ANOVA on accuracy revealed no main effects of group or sex and no interactions between sex and the other factors (all *F* < 1.27, *p* > .26, *η^2^* < .021). In contrast, the 2 x 2 x 4 ANOVA on RT revealed a significant main effect of sex (*F*(1, 60) = 4.91, *p =* .03, *η^2^* = .076) and significant group x sex (*F*(1, 60) = 4.87, *p* = .03, *η^2^* = .075) and group x sex x condition (*F*(3, 180) = 3.51, *p* = .02, *η^2^* = .055) interactions. Across groups and conditions, boys were faster to respond on correct recognition trials than girls. However, the sex x group interaction showed that boys produced faster RTs than girls only within the LR group (LR male RT < LR female RT: *p* = .004, *d* = 1.05; HR male RT < HR female RT: *p* = .99, *d* = .001). Additionally, in boys only, the LR group produced faster RTs than the HR group (LR boys < HR boys: *p* = .02, *d* = .75; LR girls < HR girls: *p* = .53, *d* = .16). Finally, the 3-way interaction between group, sex and condition showed that the faster RTs in LR boys than HR boys were restricted to the face (*p* = .01, *d* = .96) and scene (*p* = .01, *d* = 1.39) conditions but were non-significant in the car and body conditions (*p* > .06), and that the faster RTs in LR boys than LR girls were present for faces (*p* = .01, *d* = 1.29), cars (*p* = .01, *d* = 1.24) and scenes (*p* < .001, *d* = 1.38) but not for body stimuli (*p* = .34).

*Face processing ERPs:* amplitudes and latencies of the P1 and N170 components for upright and inverted faces are presented by group and sex in Table S2. The 2 x 2 x 2 x 2 ANOVA on P1 amplitude revealed a significant interaction between sex, orientation and hemisphere (*F*(1, 40) = 7.52, *p* = .009, *η^2^* = .158), but none of the planned pairwise contrasts between the levels of these factors were significant (all *p* > .10). There were no further significant main effects of sex or interactions between sex and group, orientation and hemisphere for amplitudes or latencies of the P1 or N170 (all *F* < 2.07, *p* > .16, *η^2^* < .048).

**Table S2** Means (SDs) for mid-childhood face recognition performance and face processing ERP indices presented by sex and group.

|  | **HR boys** | **HR girls** | **LR boys** | **LR girls** |
| --- | --- | --- | --- | --- |
| ***Face recognition task*** |  |  |  |  |
| *N* participants | 12 | 24 | 13 | 15 |
| Face accuracy | 68.33 (18.51) | 68.75 (16.76) | 68.46 (20.76) | 77.33 (16.24) |
| Car accuracy | 74.17 (20.21) | 68.75 (11.91) | 70.77 (18.91) | 70.67 (19.45) |
| Body accuracy | 57.50 (17.65) | 63.75 (15.27) | 64.62 (18.54) | 66.67 (14.96) |
| Scene accuracy | 71.67 (14.67) | 72.08 (18.65) | 73.08 (17.02) | 74.00 (19.20) |
| Face RT | 2374.75 (868.20) | 2443.02 (656.60) | 1720.19 (426.30) | 2356.30 (549.70) |
| Car RT | 2390.71 (911.85) | 2366.79 (907.32) | 1752.39 (418.26) | 2530.77 (784.78) |
| Body RT | 2415.46 (920.16) | 2591.15 (956.28) | 2162.04 (731.18) | 2491.97 (944.03) |
| Scene RT | 3083.08 (763.62) | 2869.13 (913.21) | 2178.96 (508.29) | 3431.27 (1155.95) |
| ***Face processing EEG task*** |  |  |  |  |
| *N* participants (P1, N170) | 7, 7 | 10, 12 | 11, 12 | 16, 14 |
| Upright P1 latency O1 | 168.23 (21.43) | 160.92 (10.06) | 174.25 (14.23) | 166.64 (20.24) |
| Upright P1 amplitude O1 | 11.37 (3.88) | 15.76 (8.63) | 11.49 (11.52) | 11.95 (8.82) |
| Upright P1 latency O2 | 159.80 (14.72) | 155.00 (14.33) | 175.44 (17.96) | 168.96 (21.22) |
| Upright P1 amplitude O2 | 12.72 (5.70) | 17.67 (8.88) | 11.50 (8.64) | 14.90 (9.98) |
| Inverted P1 latency O1 | 166.77 (18.69) | 164.20 (14.04) | 177.47 (13.96) | 175.75 (15.07) |
| Inverted P1 amplitude O1 | 11.38 (6.30) | 14.76 (9.40) | 12.84 (10.56) | 11.28 (7.57) |
| Inverted P1 latency O2 | 163.69 (17.39) | 164.56 (19.27) | 178.65 (30.23) | 172.60 (24.23) |
| Inverted P1 amplitude O2 | 12.03 (6.19) | 18.53 (9.97) | 12.23 (11.34) | 16.07 (8.80) |
| Upright N170 latency P7 | 225.51 (53.53) | 254.18 (38.08) | 252.92 (35.17) | 258.53 (40.77) |
| Upright N170 amplitude P7 | -10.09 (4.22) | -11.98 (5.81) | -7.46 (1.56) | -7.13 (2.88) |
| Upright N170 latency P8 | 254.23 (16.00) | 251.90 (25.69) | 270.18 (16.31) | 257.39 (33.03) |
| Upright N170 amplitude P8 | -15.42 (5.63) | -15.93 (6.21) | -12.50 (7.06) | -15.07 (6.96) |
| Inverted N170 latency P7 | 243.49 (42.68) | 245.97 (32.29) | 253.58 (36.63) | 247.77 (42.83) |
| Inverted N170 amplitude P7 | -10.16 (3.70) | -13.29 (5.75) | -7.96 (1.33) | -7.18 (3.07) |
| Inverted N170 latency P8 | 256.00 (14.40) | 249.88 (18.72) | 259.07 (30.77) | 246.29 (34.67) |
| Inverted N170 amplitude P8 | -15.22 (5.26) | -15.87 (6.31) | -13.10 (8.16) | -13.54 (4.55) |

*Face recognition task accuracy* = % correct trials for recognising face, car, body and scene stimuli. *Face recognition task RT* = mean of median RT (ms) for correctly recognised trials per condition. *Upright* = upright face trials. *Inverted* = inverted face trials. *O1/O2/P7/P8* = electrode locations in the left (O1/P7) and right (O2/P8) hemispheres. *Amplitude* = peak amplitude (µv) of the P1 component and the peak-to-peak amplitude (P1-N170) of the N170 component. *Latency* = latency (ms) of the peak P1 and N170 components.

**Table S3** Correlations between face recognition performance, face processing ERP indices and ASD symptoms in the HR group

|  | *7yr SRS-2* | *7yr SSP* | *7yr Face Acc* | *7yr Face RT* | *7yr N170* | *7yr P1* | *7m N290 Ampl* | *7m N290 Lat* | *7m Face looking* |
| --- | --- | --- | --- | --- | --- | --- | --- | --- | --- |
| *7yr SRS-2* | ---- |  |  |  |  |  |  |  |  |
| *7yr SSP* | **-.701**** | ---- |  |  |  |  |  |  |  |
| *7yr Face Acc* | -.109 | -.094 | ---- |  |  |  |  |  |  |
| *7yr Face RT* | **.467*** | -.208 | .070 | ---- |  |  |  |  |  |
| *7yr N170* | **-.575*** | **.716**** | -.371 | **-.672**** | ---- |  |  |  |  |
| *7yr P1* | .457 | -.461 | -.041 | .479 | -.434 | ---- |  |  |  |
| *7m N290 Ampl* | **-.425*** | .340 | .075 | -.134 | **.665*** | **-.538*** | ---- |  |  |
| *7m N290 Lat* | -.186 | .265 | -.055 | .038 | -.015 | -.213 | .074 | ---- |  |
| *7m Face looking* | -.107 | .225 | -.309 | -.176 | .209 | .081 | -.055 | -.185 | ---- |

Matrix shows the Spearman correlation coefficients between all face processing indices and ASD symptoms in the HR group. * significant at *p* < .05. ** significant with Bonferroni correction applied (*p* < .004). *7yr =* 7-year (mid-childhood) measure. *7m =* 7-month (infant) measure. *SRS-2* = Social Responsiveness Scale – Revised. *SSP* = Short Sensory Profile. *Face Acc* = accuracy (% correct trials) in the face condition of the face recognition task in mid-childhood. *Face RT* = RT (ms) for correctly recognised face trials in the face recognition task in mid-childhood. *N170* = N170 lateralisation index, representing the extent to which the amplitude (µv) of the N170 ERP component was greater in the right than left hemisphere in mid-childhood. *P1* = P1 inversion index, representing the extent to which latency (ms) of the P1 component was longer for inverted than upright face processing in mid-childhood. *N290 Ampl & Lat* = Difference scores representing the extent to which the N290 amplitude was larger and the latency was longer for processing face vs. noise stimuli at age 7 months. *Face looking* = the proportion of time spent looking at face vs. non-face stimuli in the Face Pop-Out arrays at age 7 months.

*Sensory hyper-sensitivity and hypo-sensitivity in association with neural correlates of face processing in mid-childhood*

In our main analyses we found a significant association between less right-lateralisation of the N170 component and greater sensory symptoms as measured by the SSP in high-risk siblings in mid-childhood. We conducted a post-hoc analysis to investigate whether this association held for both hyper-sensitivity and hypo-sensitivity to sensory information. Scores on the SSP subscales for tactile, movement, taste/smell and visual/auditory sensitivity were averaged to create a *hyper-sensitivity* score indexing hypersensitivity to sensory information, and the SSP under-responsiveness/seeks sensation subscale was used to index *hypo-sensitivity* to sensory information. Lower scores on these SSP scales reflect greater hyper-sensitivity and hypo-sensitivity. We computed spearman correlation coefficients between the N170 lateralisation index and the SSP hyper-sensitivity and hypo-sensitivity scores in high-risk children. This analysis showed that less right-lateralisation of the N170 was significantly associated with greater hyper-sensitivity (*rho*(17) = .627, *p* = .007) and greater hypo-sensitivity (*rho*(17) = .662, *p* = .004). These findings indicate that more atypical neural correlates of face processing in mid-childhood are associated with both hyper-sensitivity and hypo-sensitivity to sensory information in high-risk siblings.

*Longitudinal associations between N290 amplitude for face and noise stimuli and mid-childhood ASD symptoms and face processing*

In the main text we report significant associations between the N290 amplitude difference score for face vs. noise stimuli in infancy and mid-childhood SRS-2 scores and the N170 amplitude lateralisation and P1 latency inversion indices in the HR group. To better understand this pattern of findings, a supplementary post-hoc analysis was conducted to investigate whether these associations were driven by infants’ processing of face or noise stimuli or both. Spearman correlation coefficients were computed between the N290 amplitude for faces and noise and mid-childhood SRS-2 scores, N170 lateralisation index and P1 inversion index in the HR group.

The infant N290 amplitude for noise stimuli was significantly positively associated with mid-childhood SRS-2 scores (*rho*(29) = .560, *p* = .002) and the P1 lateralisation index (*rho*(14) = .609, *p* = .02) and significantly negatively correlated with the mid-childhood N170 lateralisation index (*rho*(16) = -.629, *p* = .009) (Figure S1a-c). The infant N290 amplitude for face stimuli was not associated with mid-childhood SRS-2 (*rho*(29) = .121, *p* = .53), N170 (*rho*(16) = -.238, *p* = .37) or P1 (*rho*(14) = .108, *p* = .74) measures (Figure S1a-c). This pattern of findings indicates that the longitudinal associations we report in the main text were driven by HR infants’ neural processing of noise rather than face stimuli. HR infants with smaller (less negative) N290 amplitudes to noise stimuli (which would contribute to a larger N290 difference score for faces vs. noise) had higher social-communication problems and less right-lateralised N170 components but longer P1 latencies for inverted than upright faces. These findings are discussed in the main text (section 4.2).

**Figure S1** Longitudinal associations between N290 amplitude for face and noise stimuli and mid-childhood ASD symptoms and face processing


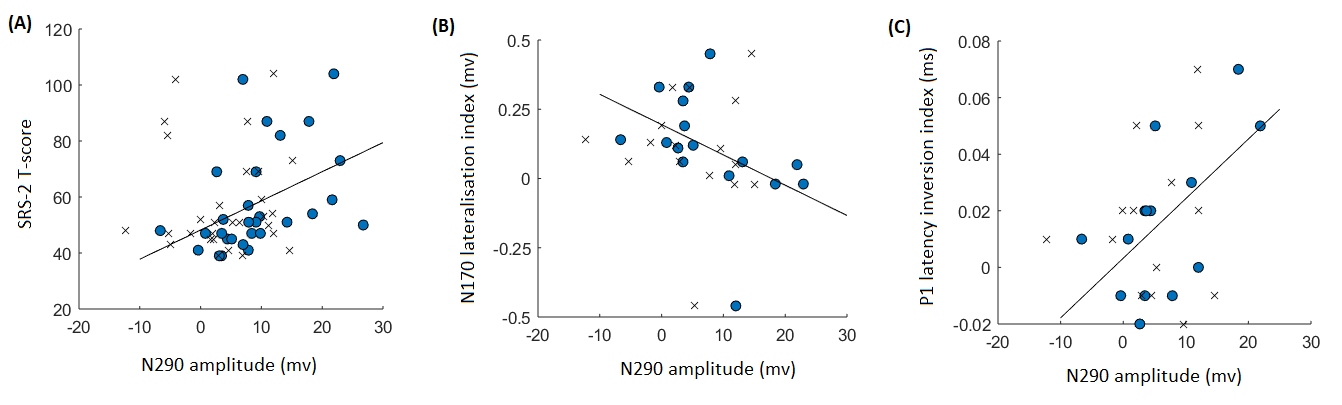


Scatterplots show the significant associations between N290 amplitude (microvolts) for noise stimuli (blue circles and regression lines) at age 7 months and mid-childhood SRS-2 scores (A), N170 amplitude lateralisation index (B) and P1 latency inversion index (C). The non-significant associations between the N290 amplitude for face stimuli at 7 months and these mid-childhood outcome variables are indicated by crosses (X). Higher SRS-2 scores indicate more severe social-communication impairments. More positive N170 lateralisation index scores indicate the amplitude for the N170 to faces in mid-childhood was larger in the right than left hemisphere. More positive P1 latency inversion index scores indicate that P1 latencies were longer for inverted than upright faces.

References

American Psychiatric Association. (2013). *Diagnostic and Statistical Manual of Mental Disorders (DSM-5)*. Washington DC: American Psychiatric Association.

Anzures, G., Goyet, L., Ganea, N., & Johnson, M. H. (2016). Enhanced ERPs to visual stimuli in unaffected male siblings of ASD children. *Child Neuropsychology*, *22*, 220-237.

Constantino, J.N. (2012). *Social Responsiveness Scale, Second Edition (SRS-2).* Los Angeles, CA: Western Psychological Services.

Dunn, W. (1999). *Short Sensory Profile*. San Antonio, TX: Psychological Corporation.

Le Couteur, A., Lord, C., & Rutter, M. (2003). *The Autism Diagnostic Interview-Revised (ADI-R).* Los Angeles, CA: Western Psychological Services.

Lord, C., Rutter, M., DiLavore, P. C., Risi, S., Gotham, K., & Bishop, S. L. (2012). *Autism Diagnostic Observation Schedule: ADOS-2*. Los Angeles, CA: Western Psychological Services.

Mullen, E. M. (1995). *Mullen Scales of Early Learning*. Circle Pines, MN: American Guidance Service.

Rutter, M., Bailey, A., & Lord, C. (2003). *The social communication questionnaire: Manual*. Los Angeles, CA: Western Psychological Services.

Shephard, E., Milosavljevic, B., Pasco, G., Jones, E. J. H., Gliga, T., Happé, F., Johnson, M. H., Charman, T. & The BASIS Team (2017). Mid-childhood outcomes of infant siblings at familial high-risk of autism spectrum disorder. *Autism Research, 10,* 546-557.

Sparrow, S. S., Balla, D. A., & Cicchetti, D. V. (2005). *Vineland Adaptive Behavior Scales, Second Edition (Vineland-II).* Bloomington, NM: Pearson Assessments.

Wechsler, D. (2011). *WASI-II: Wechsler Abbreviated Scale of Intelligence – Second Edition*. New York, NY: Psychological Corporation.
